# An Unconditionally Stable, Positivity-Preserving Splitting Scheme for Nonlinear Black-Scholes Equation with Transaction Costs

**DOI:** 10.1155/2014/525207

**Published:** 2014-05-07

**Authors:** Jianqiang Guo, Wansheng Wang

**Affiliations:** ^1^Law School, Hunan University, Hunan 410082, China; ^2^School of Mathematics and Computational Science, Changsha University of Science and Technology, Hunan 410114, China

## Abstract

This paper deals with the numerical analysis of nonlinear Black-Scholes equation with transaction costs. An unconditionally stable and monotone splitting method, ensuring positive numerical solution and avoiding unstable oscillations, is proposed. This numerical method is based on the LOD-Backward Euler method which allows us to solve the discrete equation explicitly. The numerical results for vanilla call option and for European butterfly spread are provided. It turns out that the proposed scheme is efficient and reliable.

## 1. Introduction

One of the modern financial theory's biggest successes in terms of both approach and applicability has been the Black-Scholes option pricing model developed by Black and Scholes in 1973 [1] and previously by Merton [2]:

(1) ∂V∂τ+12σ02s2∂2V∂s2+rs∂V∂s−rV=0,(s,τ)∈ΩT∶=Ω×(0,T],

where *r* > 0 and *σ*_0_ are given real constants that represent the interest rate and the volatility, respectively, *s* is the price of the underlying asset, and *T* is the maturity date. The celebrated Black-Scholes model is based on several restrictive assumptions such as liquid, frictionless, and complete markets. In recent years, nonlinear Black-Scholes models have been used to build transaction costs, market liquidity, or volatility uncertainty into the celebrated Black-Scholes concept.

In this paper, we are interested in the option pricing model with transaction costs proposed by Barles and Soner [3] that are motivated by Hodges and Neuberger [4]. In practice, transaction costs arise when trading securities. Recent studies of their influence reveal that they result in a nonnegligible increase in the option price, although they are generally small for institutional investors. To show this increase, in Barles and Soner's model, the constant volatility *σ*_0_ in the linear model is replaced by the nonlinear volatility *σ* that reads

(2) $$\begin{array}{c} \sigma^{2}=\sigma_{0}^{2}\left(1+\Psi \left(e^{r(T-\tau )}a^{2}s^{2}\frac{\partial^{2}V}{\partial s^{2}}\right)\right), \end{array}$$

where $a=\mu \sqrt{\gamma P}$ with the proportional transaction cost being *μ*, the risk aversion factor being *γ*, and the number of options to be sold being *P*, and Ψ(*x*) is the solution of the following ordinary differential equation:

(3) $$\begin{array}{c} \Psi^{\prime }\left(x\right)=\frac{\Psi \left(x\right)+1}{2\sqrt{x\Psi \left(x\right)}-x},\;x\neq 0,\;\;\Psi \left(0\right)=0. \end{array}$$

Barles and Soner's option pricing model now reads

(4) ∂V∂τ+12σ2s2∂2V∂s2+rs∂V∂s−rV=0,    (s,τ)∈ΩT∶=Ω×(0,T],

with the terminal condition

(5) $$\begin{array}{c} V\left(s,T\right)=f\left(s\right),\;s\in \Omega ∶=\left(0,+\infty \right). \end{array}$$

The payoff function *f*(*s*) is assumed to be a continuous piecewise linear function.

Many results have been reported for the numerical solutions of linear Black-Scholes equations (see, e.g., [5–9]). On the other hand, because of the nonlinear nature of this model, numerical methods are mandatory to price derivatives and portfolios. The strong nonlinearity of problem (4) makes it difficult to obtain the reliable numerical solutions. Implicit numerical schemes have been used for numerically solving nonlinear option pricing PDEs [10], and an iterative approach is required to solve the nonlinear algebraic equation resulting from the discretization, which results in more computational cost. Consequently, some high-order compact semi-implicit difference schemes are proposed for numerically solving the nonlinear option pricing model [11, 12]. Some researchers (see, e.g., [3, 13–15]) have also constructed explicit finite difference schemes for (4)-(5) and investigated their consistency and stability. However, these explicit schemes have the disadvantage that strictly restrictive conditions on the discretization parameters are needed to guarantee stability and positivity. To relax the restrictive conditions, in [16], Zhou et al. proposed an unconditionally stable explicit finite difference scheme based on a nonstandard approximation of the second partial derivative. However, this scheme is conditionally consistent, and the truncation error depends on the ratio of the time stepsize and the square of the space stepsize.

In this paper, we will consider a splitting method with inhomogeneous boundary conditions. This method proposed here is unconditionally stable, monotone, and positivity preserving. It is also consistent and essentially a “limit” version of the LOD-Backward Euler method (see Chapter IV on splitting methods in [17]) and therefore allows us to solve the discrete equation explicitly.

The paper is organized as follows: we begin by transforming the original equations into nonlinear heat equations and considering the spatial semidiscretization. The splitting scheme will be discussed in Section 3 after linearization of semidiscrete system. The stability, the monotonicity, the positivity-preserving property, and the convergence of this scheme are analysed in Section 4. To illustrate our method, we present some numerical experiments in Section 5. Finally, we give a summary.

## 2. Transformation and Spatial Semidiscretization

### 2.1. Preliminaries

We first consider the properties of the function Ψ appearing in (2), which will play an important role in the following numerical analysis.

**Lemma 1 (see [14, 15])**

The solution Ψ of ODE (3) exists and is unique, and it satisfies the following:

(1) Ψ is an increasing function mapping the real line onto the interval (−1, +*∞*);
(2) Ψ = Ψ(*x*) is implicitly defined by
  (6) x=(−
arcsinh
ΨΨ+1+Ψ)2, if  Ψ>0,x=−(
arcsinh
−ΨΨ+1−−Ψ)2, if  −1<Ψ<0;
(3) if *x* > 0, then the function Ψ(*x*) is bounded and
  (7) 0<Ψ(x)<Ψ′(x2)x+d2,
  where

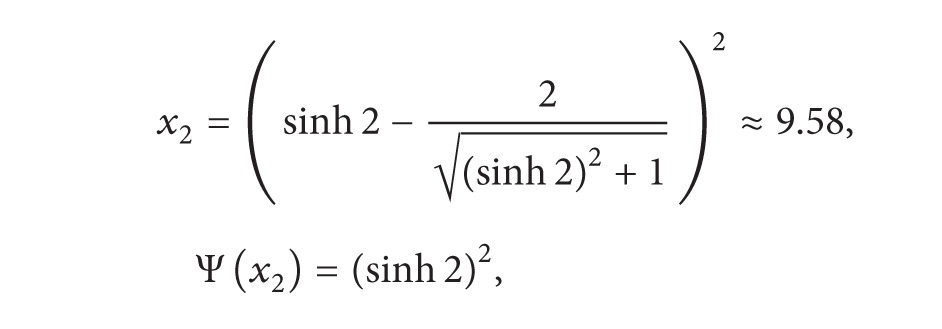
(8)

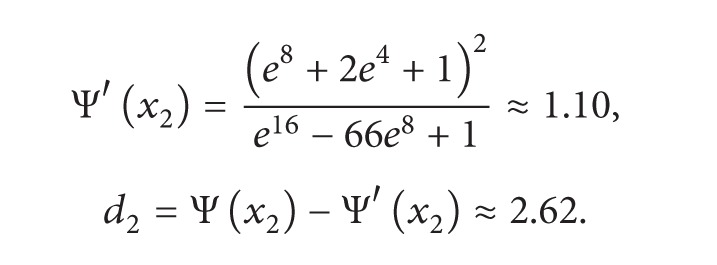
(9)

**Lemma 2 (see [14, 15])**

The function *g*(*x*) = *x*Ψ(*x*) is continuously differentiable at *x* = 0 and satisfies

(10) $$\begin{array}{c} \left|g^{\prime }\left(x\right)\right|\leq \max\left\{G,2\left|x\right|\Psi^{\prime }\left(x_{2}\right)+d_{2}\right\}, \end{array}$$

where *x*_2_ and *d*_2_ are given by (8) and (9), respectively, and

(11) $$\begin{array}{c} G=\max\left\{\left|g^{\prime }\left(x\right)\right|;x_{1}\leq x\leq x_{2}\right\},\;\;x_{1}=-\frac{{\left(4\pi -3\sqrt{3}\right)}^{2}}{36}. \end{array}$$

Combining Lemma 2 and the results obtained in [3] leads to the following lemma.

**Lemma**

The function *w*(*x*) = *x* + *x*Ψ(*x*) is a nondecreasing function of *x* and continuously differentiable at *x* = 0. This implies that

(12) $$\begin{array}{c} 1+\Psi \left(x\right)+x\Psi^{\prime }\left(x\right)=\frac{d}{dx}\left[x+x\Psi \left(x\right)\right]\geq 0. \end{array}$$

### 2.2. The Transformed Problem

After considering the change of variable

(13) $$\begin{array}{c} S=e^{r(T-\tau )}s,\;\;t=T-\tau ,\;\;U\left(S,t\right)=e^{r(T-\tau )}V\left(s,\tau \right), \end{array}$$

we transform the problem (4)-(5) into the following initial value problem

(14) ∂U∂t−σ02S22(1+Ψ(a2S2∂2U∂S2))∂2U∂S2=0,(S,t)∈Ω×(0,T],

(15) $$\begin{array}{c} U\left(S,0\right)=f\left(S\right),\;S\in \Omega . \end{array}$$

The boundary conditions take the form of

(16) $$\begin{array}{c} U\left(0,t\right)=0,\;U\left(S,t\right)=\alpha \left(+\infty ,t\right),\;t\in \left[0,T\right], \end{array}$$

where the second boundary condition in (16) is derived by asymptotic considerations in (*s*, *τ*) coordinates for extreme value *s* → *∞* and subsequent transformation. For example, the boundary conditions for a vanilla call are given by

(17) $$\begin{array}{c} U\left(0,t\right)=0,\;\underset{S\to +\infty }{\lim}\frac{\alpha \left(S,t\right)}{S}=1,\;t\in \left[0,T\right], \end{array}$$

and the boundary conditions for a butterfly spread are given by

(18) $$\begin{array}{c} U\left(0,t\right)=0,\;\underset{S\to +\infty }{\lim}\alpha \left(S,t\right)=0,\;t\in \left[0,T\right]. \end{array}$$

### 2.3. The Semidiscrete Nonlinear System

To numerically approximate the solution of (14)–(16), we should consider a bounded numerical domain (*S*, *t*)∈[0, *b*]×[0, *T*]. Then, the problem (14)-(15) is equipped with the boundary conditions

(19) $$\begin{array}{c} U\left(0,t\right)=0,\;U\left(b,t\right)=\alpha \left(b,t\right),\;t\in \left[0,T\right]. \end{array}$$

Note that the second equality in (19) derives from the second equality in (16). Taking these into consideration, the boundary conditions for a vanilla call are given by

(20) U(0,t)=0, U(b,t)=α(b)=max⁡{0,b−E},t∈[0,T],

where *E* is the strike price, and the boundary conditions for a butterfly spread are given by

(21) $$\begin{array}{c} U\left(0,t\right)=0,\;U\left(b,t\right)=\alpha \left(b\right)=f\left(b\right),\;\;t\in \left[0,T\right]. \end{array}$$

We introduce the spatial grid *Ω*_*h*_ with step *h* by the nodes *S*_*i*_ = *ih*, *i* = 0,1,…, *M*, so that *Mh* = *b*. After performing the second-order central finite difference approximation of the partial derivative (∂^2^ *U*/∂*S* ^2^)(*S*, *t*) as

(22) ∂2U∂S2=U(Si+1,t)−2U(Si,t)+U(Si−1,t)h2+O(h2)=ΔiU(t)+O(h2),

we obtained the corresponding ODEs system for the semidiscrete solution *u*(*t*) = [*U*_1_(*t*),*U*_2_(*t*),…,*U*_*M*−1_(*t*)]^*T*^ as

(23) $$\begin{array}{c} u^{\prime }\left(t\right)=A\left(u\left(t\right)\right)u\left(t\right)+g\left(u\left(t\right)\right),\;t\in \left[0,T\right], \end{array}$$

with

(24) $$\begin{array}{c} A\left(u\right)=\frac{1}{h^{2}}\text{tridiag}\left(\beta_{i}\left(u\right),-2\beta_{i}\left(u\right),\beta_{i}\left(u\right)\right),\\ \beta_{i}\left(u\right)=\sigma_{i}^{2}\left(u\right)S_{i}^{2},\\ \sigma_{i}^{2}\left(u\right)=\frac{1}{2}\sigma_{0}^{2}\left(1+\Psi_{i}\right),\\ \Psi_{i}=\Psi \left(a^{2}S_{i}^{2}\Delta_{i}u\left(t\right)\right), \end{array}$$

where *g* ∈ *R* ^*M*−1^ is a vector, generated by the boundary conditions,

(25) $$\begin{array}{c} g\left(u\left(t\right)\right)=\frac{1}{h^{2}}{\left[0,0,\ldots ,0,\beta_{M-1}\alpha \left(b\right)\right]}^{T}. \end{array}$$

Obviously, the semidiscrete difference scheme (23) is consistent. For the stability, we have the following theorem.

**Theorem 4**

For system (23), the following maximum norm contractivity holds:

(26) $$\begin{array}{c} {||u(t)-\tilde{u}(t)||}_{\infty }\leq {||u(0)-\tilde{u}(0)||}_{\infty },\;\forall t\in \left[0,T\right], \end{array}$$

where $\tilde{u}$ is the solution of the perturbation problem

(27) $$\begin{array}{c} {\tilde{u}}^{\prime }\left(t\right)=A\left(\tilde{u}\left(t\right)\right)\tilde{u}\left(t\right)+g\left(\tilde{u}\left(t\right)\right),\;t\in \left[0,T\right], \end{array}$$

with the initial data $\tilde{u}(0)$.

**Proof**

To obtain the stability of the semidiscrete difference scheme (23), we first calculate

(28) $$\begin{array}{c} \frac{\partial \beta_{i}}{\partial U_{i}}=\frac{1}{2}\sigma_{0}^{2}S_{i}^{2}\Psi_{i}^{\prime }\cdot \left(\frac{-2a^{2}S_{i}^{2}}{h^{2}}\right)=-\frac{1}{h^{2}}\sigma_{0}^{2}a^{2}S_{i}^{4}\Psi_{i}^{\prime };\\ \frac{\partial \beta_{i}}{\partial U_{i-1}}=\frac{\partial \beta_{i}}{\partial U_{i+1}}=\frac{1}{2}\sigma_{0}^{2}S_{i}^{2}\Psi_{i}^{\prime }\cdot \left(\frac{a^{2}S_{i}^{2}}{h^{2}}\right)=\frac{1}{2h^{2}}\sigma_{0}^{2}a^{2}S_{i}^{4}\Psi_{i}^{\prime }. \end{array}$$

Now, let nonlinear operator *F*(*u*) be defined by *F*(*u*) = *A*(*u*)*u* + *g*(*u*). For system (23), we have

(29) ∂Fi∂Ui=1h2(−2βi−2∂βi∂UiUi+∂βi∂Ui(Ui−1+Ui+1))=1h2(−2βi−Ui−1−2Ui+Ui+1h2σ02a2Si4Ψi′)=−1h2(2βi+Δiu(t)σ02a2Si4Ψi′),∂Fi∂Ui−1=∂Fi∂Ui+1=1h2(βi+Ui−1−2Ui+Ui+1h212σ02a2Si4Ψi′)=12h2(2βi+Δiu(t)σ02a2Si4Ψi′).

Since

(30) 2βi+Δiu(t)σ02a2Si4Ψi′=1+Ψi(a2Si2Δiu(t)) +a2Si2Δiu(t)Ψi′(a2Si2Δiu(t)),

by Lemma 3, we obtain

(31) $$\begin{array}{c} \frac{\partial F_{i}}{\partial U_{i}}\leq 0,\;\;\frac{\partial F_{i}}{\partial U_{i-1}}=\frac{\partial F_{i}}{\partial U_{i+1}}\geq 0. \end{array}$$

Then, inequality (26) follows from *μ*_*∞*_[*J*_*F*_(*u*)] = 0 [18], where *J*_*F*_(*u*) is the Jacobian matrix of *F* and *μ*_*∞*_[·] is the logarithmic maximum norm.

## 3. Splitting Time-Stepping Method

In this section, we will focus on the time integration methods of system (23). The implicit numerical schemes are generally viewed as stable methods, but [10] showed that the stability of the BDF schemes with orders 1 and 2 and the Crank-Nicolson scheme is still restricted by a condition. Additionally, for the implicit schemes, the nonlinear iteration will be required and will produce the additional computational cost in each time step. Fully explicit finite difference schemes for the PDEs (14)-(15) are also constructed in [3, 13–15]. However, these schemes are stable only for the severe restriction on the time stepsize Δ*t*. For example, in [14], the condition reads

(32) $$\begin{array}{c} \frac{\Delta t}{h^{3}}\left[\left(1+d_{2}\right)h+4a^{2}E^{2}\Psi^{\prime }\left(x_{2}\right)\right]\leq \frac{1}{8E^{2}}. \end{array}$$

To relax the condition, in this paper, we will propose an unconditionally stable method, which allows us to solve the discrete equation explicitly, based on the LOD-Backward Euler method (see Chapter IV on splitting methods in [17]).

### 3.1. Linearization System

Let us set *t*_*n*_ = *n*Δ*t*, *n* = 1,2,…, *N*, for the temporal stepsize Δ*t* = *T*/*N*. To linearize the nonlinear system (23), we allow the nonlinearities in (23) to lag one step behind and obtain the following linear system:

(33) u′(t)=A(u(tn))u(t)+g(u(tn))=Anu(t)+gn,t∈[tn,tn+1],

with

(34) An=1h2tridiag(βin,−2βin,βin),gn=1h2[0,0,…,0,βM−1nα(b)]T,

where

(35) $$\begin{array}{c} \beta_{i}^{n}=\sigma_{i,n}^{2}S_{i}^{2},\;\;\sigma_{i,n}^{2}=\frac{1}{2}\sigma_{0}^{2}\left(1+\Psi_{i}^{n}\right),\\ \Psi_{i}^{n}=\Psi \left(a^{2}S_{i}^{2}\Delta_{i}U^{n}\right),\\ \Delta_{i}U^{n}=\frac{U_{i-1}^{n}-2U_{i}^{n}+U_{i+1}^{n}}{h^{2}},\;U^{n}={\left[U_{1}^{n},U_{2}^{n},\ldots ,U_{M-1}^{n}\right]}^{T}. \end{array}$$

For this linearized system, we have

(36) $$\begin{array}{c} u\left(t\right)=e^{(t-t_{n})A_{n}}u\left(t_{n}\right)+\overset{t}{\underset{t_{n}}{\int }}e^{(t-s)A_{n}}g_{n}ds, \end{array}$$

and therefore

(37) $$\begin{array}{c} u\left(t\right)=e^{(t-t_{n})A_{n}}u\left(t_{n}\right)+\left[e^{(t-t_{n})A_{n}}-I\right]A_{n}^{-1}g_{n}. \end{array}$$

### 3.2. Numerical Method Construction

In this subsection, we construct a splitting time-stepping method based on (37). In this paper, for matrices *C*_*i*_, we define

(38) $$\begin{array}{c} \overset{M-1}{\underset{i=1}{\prod }}C_{i}∶=C_{M-1}C_{M-2}\cdots C_{2}C_{1}. \end{array}$$

Then, the following is not true in general:

(39) $$\begin{array}{c} \overset{M-1}{\underset{i=1}{\prod }}C_{i}=C_{1}C_{2}\cdots C_{M-2}C_{M-1}. \end{array}$$

Let us consider the following splitting:

(40) $$\begin{array}{c} A_{n}=\overset{M-1}{\underset{i=1}{\sum }}A_{n,i},\;\;g_{n}=\overset{M-1}{\underset{i=1}{\sum }}g_{n,i}, \end{array}$$

with

(41) An,1=1h2[−2β1nβ1n⋯000⋯0⋮⋮⋱⋮00⋯0],An,M−1=1h2[00⋯0000⋯00⋮⋮⋱⋮⋮00⋯0000⋯βM−1n−2βM−1n],An,i=1h2[0⋯⋯⋯⋯⋯00⋯⋯⋯⋯⋯0⋮⋮⋱⋮⋮⋮⋮0⋯βin−2βinβin⋯0⋮⋮⋮⋮⋱⋮⋮0⋯⋯⋯⋯⋯00⋯⋯⋯⋯⋯0],i=2,3,…,M−2,gn,i=0, i=1,2,…,M−2,gn,M−1=1h2[0,0,…,0,βM−1nα(b)]T,

where 0 ∈ *R* ^*M*−1^ is the zero vector. Obviously, other choices for *g*_*n*,*i*_ are possible; for example, *g*_*n*,1_ = *g*_*n*_, *g*_*n*,*i*_ = 0,  *i* = 2,3,…, *M* − 1.

With the splitting (40), we solve the *M* − 1 subproblems:

(42) dui(t)dt=An,iui(t)+gn,i, ui(tn)=ui−1(tn),i=1,2,…,M−1,

starting from *u* ^0^(*t*_*n*_) = *u*(*t*_*n*_), and we take *u*(*t*_*n*+1_) = *u* ^*M*−1^(*t*_*n*+1_) to complete the splitting integration step.

Then, if the Backward Euler method is used to solve every subproblem, we get

(43) $$\begin{array}{c} u_{n}^{i}={\left[I-\Delta tA_{n,i}\right]}^{-1}u_{n}^{i-1}+{\left[I-\Delta tA_{n,i}\right]}^{-1}\Delta tg_{n,i}, \end{array}$$

where *u*_*n*_^*i*^ is an approximation of *u* ^*i*^(*t*_*n*_). It is easily verified that the matrix *I* − Δ*tA*_*n*,*i*_ is *M*-matrix and therefore is nonsingular. By induction, we have

(44) un+1=un+10=unM−1=∏i=1M−1[I−ΔtAn,i]−1un +∑j=1M−1∏i=j+1M−1[I−ΔtAn,i]−1[I−ΔtAn,j]−1Δtgn,j,

which implies that for the splitting (41)

(45) $$\begin{array}{c} u_{n+1}=\overset{M-1}{\underset{i=1}{\prod }}{\left[I-\Delta tA_{n,i}\right]}^{-1}u_{n}+{\left[I-\Delta tA_{n,M-1}\right]}^{-1}\Delta tg_{n,M-1}. \end{array}$$

For other choices of *g*_*n*,*i*_, we can similarly obtain corresponding splitting schemes.

By brief calculation, one can obtain an explicit expression of matrix [*I*−Δ*tA*_*n*,*i*_]^−1^:

(46) [I−ΔtAn,1]−1=[h2h2+2Δtβ1nΔtβ1nh2+2Δtβ1n⋯001⋯0⋮⋮⋱⋮00⋯1],[I−ΔtAn,i]−1=[10⋯000⋯0001⋯000⋯00⋮⋮⋱⋮⋮⋮⋮⋮⋮00⋯100⋯0000⋯Δtβinh2+2Δtβinh2h2+2ΔtβinΔtβinh2+2Δtβin⋯0000⋯001⋯00⋮⋮⋮⋮⋮⋮⋱⋮⋮00⋯000⋯1000⋯000⋯01],i=2,3,⋯,M−2,[I−ΔtAn,M−1]−1 =[10⋯0001⋯00⋮⋮⋱⋮⋮00⋯1000⋯ΔtβM−1nh2+2ΔtβM−1nh2h2+2ΔtβM−1n].

Then, we get the following explicit formula for computing *u*_*n*+1_ = [*U*_1_^*n*+1^, *U*_2_^*n*+1^,…, *U*_*M*−1_^*n*+1^]^*T*^, where *U*_*i*_^*n*^ is an approximation of *U*(*S*_*i*_, *t*_*n*_):

(47) U1n+1=h2h2+2Δtβ1nU1n+Δtβ1nh2+2Δtβ1nU2n,Uin+1=Δtβinh2+2ΔtβinUi−1n+1+h2h2+2ΔtβinUin+Δtβinh2+2ΔtβinUi+1n,i=2,3,…,M−2,UM−1n+1=ΔtβM−1nh2+2ΔtβM−1nUM−2n+1+h2h2+2ΔtβM−1nUM−1n +ΔtβM−1nh2+2ΔtβM−1nα(b).

For the finite-dimensional discrete system (33), the splitting in the numerical scheme (45) is such that all computations become effectively “truly” one-dimensional. The numerical scheme (45) can be viewed as a “limit” version of LOD-Backward Euler method (see Chapter IV on splitting methods in [17]).

## 4. Properties of the Numerical Scheme

In this section, we investigate some properties of the numerical scheme proposed here.

### 4.1. Stability

In [17], the stability of LOD-Backward Euler method is provided. The following theorem shows the stability of the numerical scheme (45).

**Theorem**

The numerical scheme (45) is unconditionally stable in both the spectral norm ||·|| and the maximum norm ||·||_*∞*_; that is, one has the following stability inequalities:

(48) $$\begin{array}{c} ||u_{n}-{\tilde{u}}_{n}||\leq ||u_{0}-{\tilde{u}}_{0}||,\;\forall n\geq 0, \end{array}$$

(49) $$\begin{array}{c} {||u_{n}-{\tilde{u}}_{n}||}_{\infty }\leq {||u_{0}-{\tilde{u}}_{0}||}_{\infty },\;\forall n\geq 0. \end{array}$$

It should be pointed out that (49) can be regarded as a discrete version of maximum norm contractivity (26).

**Proof**

To obtain the spectral norm stability inequality (48), we apply the Gerschgorin theorem to the matrix *A*_*n*,*i*_ and get that the nonzero matrix eigenvalues line in the disc

(50) $$\begin{array}{c} \left|z+2\sigma_{i,n}^{2}S_{i}^{2}\right|\leq 2\sigma_{i,n}^{2}S_{i}^{2}, \end{array}$$

and therefore they are nonpositive. Then, any of the eigenvalues *ζ*_*i*_ of the matrix [*I* − Δ*tA*_*n*,*i*_]^−1^, corresponding to the eigenvalues *η*_*i*_ of *A*_*n*,*i*_, satisfies

(51) $$\begin{array}{c} \left|\zeta_{i}\right|\leq \frac{1}{\left|1-\Delta t\eta_{i}\right|}\leq 1. \end{array}$$

Thus, it is easy to obtain ∏_*i*=1_^*M*−1^ | *ζ*_*i*_ | ≤1 and *ρ*(∏_*i*=1_^*M*−1^[*I* − Δ*tA*_*n*,*i*_]^−1^) ≤ 1, where *ρ*(·) denotes the spectral radius of the matrix. The stability inequality (48) follows from this estimate.

We note that

(52) $$\begin{array}{c} \mu_{\infty }\left[A_{n,i}\right]\leq 0,\;i=1,2,\ldots ,M-1, \end{array}$$

which directly leads to the maximum norm stability inequality (49) (see [17]).

### 4.2. Positivity

A nice property of the numerical scheme for the pricing equation is positivity preserving, since the value of option is nonnegative.

**Theorem**

The numerical scheme (45) is unconditionally positivity preserving; that is, the solution of (45) is positive on each time level *t*_*n*+1_, *n* = 0,1,…, *N* − 1, if *u*_0_ is positive.

**Proof**

Since all entries of the matrices [*I*−Δ*tA*_*n*,*i*_]^−1^, *i* = 1,2,…, *M* − 1, are nonnegative, a nonnegative solution *u*_*n*+1_ of (45) on each time level can be obtained in view of the nonnegative property of *g*_*n*,*i*_ and *u*_0_. This completes the proof.

### 4.3. Monotonicity

Let us now consider the monotonicity of the numerical scheme (45). To do this, we note that scheme (45) can be written as

(53) $$\begin{array}{c} U_{i}^{n+1}=H\left(U_{i-1}^{n},U_{i}^{n},U_{i+1}^{n}\right),\;i=1,2,\ldots ,M-1. \end{array}$$

For scheme (53), we have the following definition of monotonicity.

**Definition (see [19]; see also [20, 21])**

Scheme (53) is said to be monotone if and only if *H* is nondecreasing in each argument.

The following theorem shows the monotonicity of the numerical scheme (45).

**Theorem**

The numerical scheme (45) is unconditionally monotone.

**Proof**

From the proof of Theorem 6, it is known that all entries of the matrices [*I*−Δ*tA*_*n*,*i*_]^−1^, *i* = 1,2,…, *M* − 1, are nonnegative. Then, *H* is nondecreasing in each argument, and therefore the scheme is monotone.

### 4.4. Maximum Principle

From (43) and (46), it is not difficult to obtain

(54) min⁡{Ui−1n,Uin,Ui+1n}≤Uin+1≤max⁡{Ui−1n,Uin,Ui+1n},i=1,2,…,M−1.

Then, we have the maximum principle

(55) min⁡j{Uj0}≤Uin≤max⁡j{Uj0},i=1,2,…,M−1, ∀n≥0,

which also implies maximum norm estimate; that is, ||*u*_*n*_||_*∞*_ ≤ ||*u*_0_||_*∞*_.

### 4.5. Local Error Analysis and Consistency

Now, we consider the error. Let $\epsilon_{n}=u(t_{n})-{\tilde{u}}_{n}$ denote the local discretization error, that is, the error introduced in one single step of the method. To bound the local discretization errors *ϵ*_*n*_, we need the following truncation error estimation.

**Lemma (see [22])**

For splitting *A*_*n*_ = ∑_*i*=1_^*M*−1^ *A*_*n*,*i*_, one has

(56) [eΔtAn−∏i=1M−1eΔtAn,i]u(tn)  =Δt22∑i=1M−1∑j=i+1M−1[An,i,An,j]u(tn)+O(Δt3),

with

(57) $$\begin{array}{c} \left[A_{n,i},A_{n,j}\right]=A_{n,i}A_{n,j}-A_{n,j}A_{n,i} \end{array}$$

being the commutator of *A*_*n*,*i*_ and *A*_*n*,*j*_, and

(58) $$\begin{array}{c} \left[e^{\Delta tA_{n}}-\frac{1}{2}\left(\overset{M-1}{\underset{i=1}{\prod }}e^{\Delta tA_{n,i}}+\overset{M-1}{\underset{i=1}{\prod }}e^{\Delta tA_{n,M-i}}\right)\right]u\left(t_{n}\right)=O\left(\Delta t^{3}\right). \end{array}$$

**Theorem 10**

With the previous notation, the local discretization error *ϵ*_*n*_ of the numerical scheme (45) is given by

(59) $$\begin{array}{c} \epsilon_{n}=O\left(\Delta t^{2}\right). \end{array}$$

**Proof**

It follows from (37) that

(60) u(tn+1)=eΔtAnu(tn)+[eΔtAn−I]An−1gn=∏i=1M−1eΔtAn,iu(tn)+[eΔtAn−∏i=1M−1eΔtAn,i]u(tn) +[eΔtAn−I]An−1gn.

Because of the expansion formula

(61) $$\begin{array}{c} e^{\Delta tA_{n}}=\overset{\infty }{\underset{k=0}{\sum }}\frac{1}{k!}{\left(\Delta tA_{n}\right)}^{k}=I+\Delta tA_{n}+\frac{1}{2}{\left(\Delta t\right)}^{2}A_{n}^{2}+\cdots , \end{array}$$

we have

(62) [eΔtAn−I]An−1gn  =Δt∑k=0∞1(k+2)!(ΔtAn)kgn  =Δt∑i=1M−1(I+12ΔtAn+16(Δt)2An2+⋯)gn,i.

We also have the following expansion formula similar to (61):

(63) eΔtAn,i=∑k=0∞1k!(ΔtAn,i)k=I+ΔtAn,i+12(Δt)2An,i2+16(Δt)3An,i3+⋯,i=1,2,…,M−1.

It is easy to obtain the expansion formula of [*I*−Δ*tA*_*n*,*i*_]^−1^, *i* = 1,2,…, *M* − 1:

(64) [I−ΔtAn,i]−1=I+∑k=1∞(ΔtAn,i)k=I+ΔtAn,i+(Δt)2An,i2+(Δt)3An,i3+⋯.

Comparing (63) with (64) yields, for *i* = 1,2,…, *M* − 1,

(65) $$\begin{array}{c} e^{\Delta tA_{n,i}}={\left[I-\Delta tA_{n,i}\right]}^{-1}-\frac{1}{2}{\left(\Delta t\right)}^{2}A_{n,i}^{2}+\cdots , \end{array}$$

which implies that

(66) $$\begin{array}{c} \overset{M-1}{\underset{i=1}{\prod }}e^{\Delta tA_{n,i}}=\overset{M-1}{\underset{i=1}{\prod }}{\left[I-\Delta tA_{n,i}\right]}^{-1}-\frac{1}{2}\overset{M-1}{\underset{i=1}{\sum }}{\left(\Delta t\right)}^{2}A_{n,i}^{2}+\cdots . \end{array}$$

From (63) and (62), one gets

(67) [eΔtAn−I]An−1gn  =Δt∑i=1M−1(∏j=iM−1[I−ΔtAn,j]−1+∑j=1i−112ΔtAn,j‍−∑j=iM−112ΔtAn,j+O(Δt2An2))gn,i.

For splitting (41), since *g*_*n*,*i*_ = 0, *i* = 1,2,…, *M* − 2, we have

(68) [eΔtAn−I]An−1gn  =([I−ΔtAn,M−1]−1+∑j=1M−212ΔtAn,j−12ΔtAn,M−1+O(Δt2An2))Δtgn,M−1.

Substituting (66) and (68) into (60) obtains

(69) u(tn+1) =∏i=1M−1[I−ΔtAn,i]−1u(tn)+[eΔtAn−∏i=1M−1eΔtAn,i]u(tn)  +[eΔtAn−I]An−1gn+O(Δt2) =u~n+1−12Δt2An,M−1gn,M−1  +∑j=1M−212Δt2An,jgn,M−1+O(Δt2)  +[eΔtAn−∏i=1M−1eΔtAn,i]u(tn).

Then, (59) follows from the above equation and (56).

We can now immediately conclude that the splitting scheme (45) is consistent.

### 4.6. Viscosity Solutions and Convergence

The so-called viscosity solutions are the meaningful solutions in financial applications. This has been shown in [23, 24]. Putting all results together, we can formulate the following convergence result.

**Theorem**

The splitting Backward Euler scheme (45) unconditionally converges to the viscosity solution of (14)-(15).

**Proof**

The convergence of the fully-discrete scheme (45) is a direct result of the consistency, the stability, and the monotonicity of this scheme [25].

## 5. Numerical Experiments

In order to illustrate the stability and convergence properties of our proposed scheme, in this section, we present several numerical experiments in which the vanilla call option and the European butterfly spread are considered. To obtain the numerical solution of nonlinear Black-Scholes equations (14)–(16), we should first solve the ODE (3). In this paper, we use the symmetric midpoint scheme to solve numerically the ODE (3). The utility function Ψ(*x*) can be done by linear interpolation.

**Example**

Let us consider a vanilla European call option with

(70) $$\begin{array}{c} f\left(s\right)=\max\left(s-E,0\right). \end{array}$$

**Figure 1:**
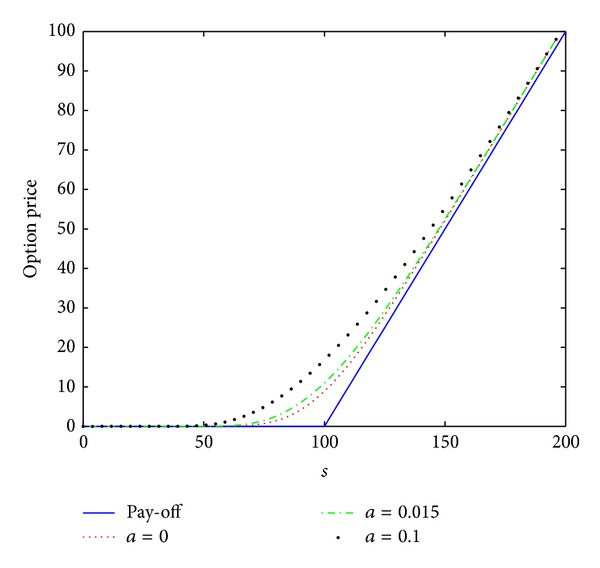
Option pricing of a vanilla European call option for several values of parameter *a* at maturity time *T* = 1 year, where *h* = 4 and Δ*t* = 0.0002.

This example comes from [14]. The parameters are the following: the strike price *E* = 100, the volatility *σ*_0_ = 0.2, the interest rate *r* = 0.02, the maturity date *T* = 1 year, and the artificial boundary location *b* = 200. We first consider a small time stepsize Δ*t* = 1/*N* = 0.0002 and a spatial stepsize *h* = 200/*M* = 4 as done in [14]. The numerical results *V*(*s*, *τ*) computed by the numerical scheme (45) are plotted in Figure 1 in (*s*, *τ*) coordinate. The case of *a* = 0 is linear model, and the cases of *a* = 0.015 and *a* = 0.1 are nonlinear model. The numerical results are the same as those showed in [14].

**Figure 2:**
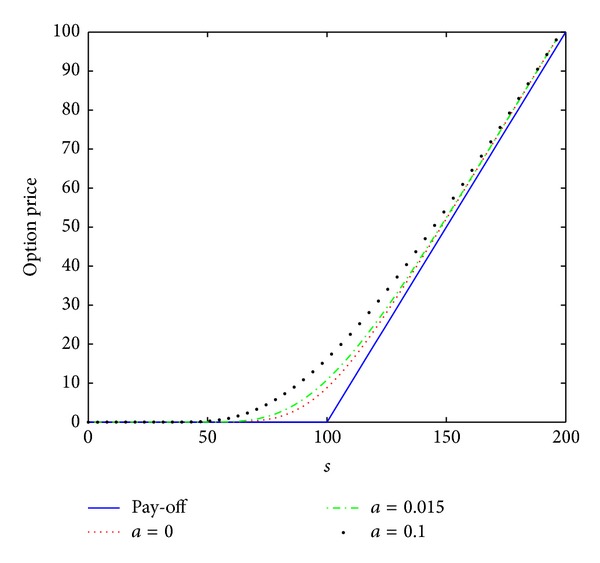
Option pricing of a vanilla European call option for several values of parameter *a* at maturity time *T* = 1 year, where *h* = 4 and Δ*t* = 0.002.

**Figure 3:**
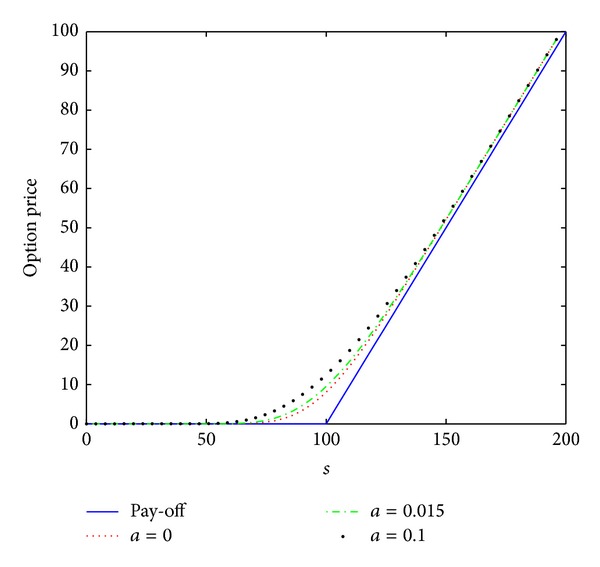
Option pricing of a vanilla European call option for several values of parameter *a* at maturity time *T* = 1 year, where *h* = 4 and Δ*t* = 0.02.

To further confirm that this scheme is unconditionally stable, monotone, and positivity preserving, we also calculate the numerical solutions, which are presented in Figures 2 and 3, by scheme (45) with larger time stepsizes Δ*t* = 0.002 and Δ*t* = 0.02, respectively. From Figures 2 and 3, we observe that there are no stability issues for scheme (45). We also note that the scheme proposed in [14] produces the wrong numerical solution when the stepsize Δ*t* = 0.00027027 such that the stability condition (32) is not satisfied (see [14]). This reveals that the numerical scheme (45) has better stability properties than the scheme proposed in [14].

To illustrate the convergence of scheme (45), we show the option value at *S* = *E* = 100 (or at *s* = 98.01987 at time to maturity being 1 year), the differences between successive approximation processes, and the ratios between the differences in Table 1. Since scheme (45) is of order two in space and order one in time, the number of spatial mesh points is double, but the number of temporal grid points is quadruple. Table 1 only shows the numerical results for the case *a* = 0.015, which confirm our theoretical analysis results. The numerical results for the cases of *a* = 0 and *a* = 0.1 are similar and we omit these here.

From the data presented in Figures 1–3 and Table 1, the conclusion can be drawn: scheme (45) is unconditionally stable and convergent.

**Example (european butterfly spread)**

This example comes from [16]. A butterfly spread consists of two long positions in two calls with *E*_1_, *E*_2_ and a short position in two calls at strike *S* = (*E*_1_ + *E*_2_)/2. The payoff function *f*(*S*) is given by

(71) $$\begin{array}{c} f\left(S\right)=\max\left(S-E_{1},0\right)-2\max\left(S-E,0\right)+\max\left(S-E_{2},0\right). \end{array}$$

**Figure 4:**
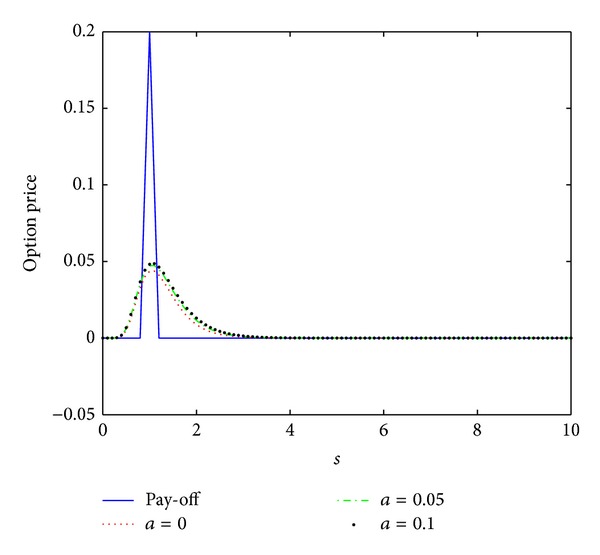
Option pricing of a European butterfly spread for several values of parameter *a* at maturity time *T* = 0.5, where *h* = 0.1 and Δ*t* = 0.0005.

**Figure 5:**
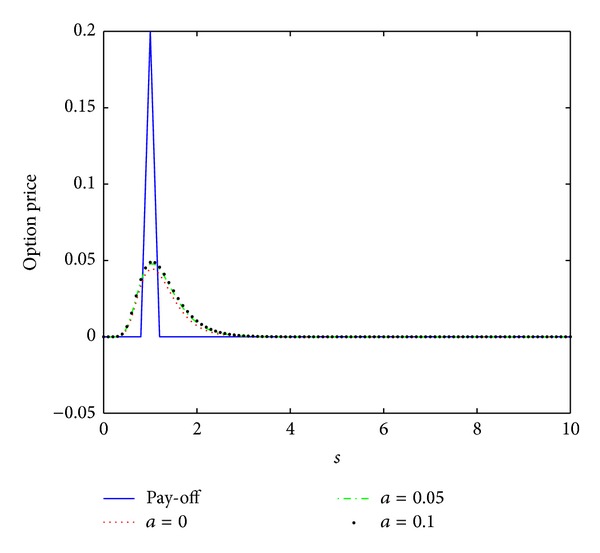
Option pricing of a European butterfly spread for several values of parameter *a* at maturity time *T* = 0.5, where *h* = 0.1 and Δ*t* = 0.005.

**Figure 6:**
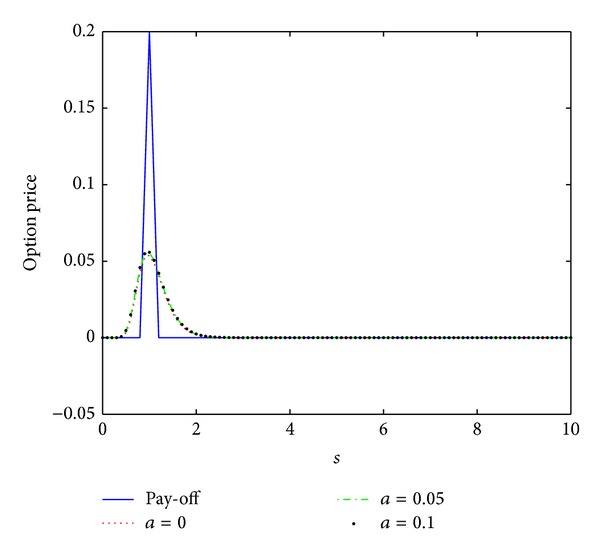
Option pricing of a European butterfly spread for several values of parameter *a* at maturity time *T* = 0.5, where *h* = 0.1 and Δ*t* = 0.05.

Let *E*_1_ = 0.8, *E*_2_ = 1.2, the volatility *σ*_0_ = 0.5, the interest rate *r* = 0.04, the maturity date *T* = 0.5, and the artificial boundary location *b* = 10. We first compute the numerical solution by scheme (45) with the time stepsize Δ*t* = 0.5/*N* = 0.0005 and the spatial stepsize *h* = 10/*M* = 0.1 similar to those in [16]. The numerical results are presented in Figure 4. These numerical data together with those in Figures 5 and 6 show that this scheme proposed here is unconditionally stable.

In [16], the authors compared the numerical results obtained by their nonstandard scheme and the schemes proposed in [14, 15] and showed that their scheme produces better numerical solutions than the schemes in [14, 15] with the same stepsizes. To compare our scheme with the nonstandard scheme proposed in [16], we first observe that both of the numerical schemes are unconditionally stable. Then, we will investigate their convergence and compare their convergence order. To this end, we calculate the discrete maximum norms of the errors

(72) $$\begin{array}{c} E_{\infty }^{\text{Sp}}=\underset{i}{\max}||U_{i}^{N,\text{Sp}}-{\tilde{U}}_{i}^{N}||,\;\;E_{\infty }^{\text{Ns}}=\underset{i}{\max}||U_{i}^{N,\text{Ns}}-{\tilde{U}}_{i}^{N}||, \end{array}$$

where *U*_*i*_^*N*,Sp^ and *U*_*i*_^*N*,Ns^ denote the numerical solutions computed by our splitting scheme (45) and the nonstandard scheme proposed in [16] at the maturity date *T* = 0.5, respectively, and ${\tilde{U}}_{i}^{N}$ denotes the numerical reference solution computed by the Backward Euler method with the standard second-order finite differences on a finer grid with *N* = 2560 and *M* = 320. Numerical results of both schemes (45) and the nonstandard scheme proposed in [16] are listed in Table 2. This table shows the maximum norm of the errors and the ratios between these errors. It is evident from the numerical data obtained by the splitting scheme (45) that this scheme is convergent with the temporal order one and the spatial order two. This is consistent with the theoretical results presented in this paper. For the nonstandard scheme proposed in [16], however, a reduction in the error is observed. This is not surprising since, as Zhou et al. pointed out in [16], the nonstandard scheme is conditionally consistent and the truncation error really depends on the ratio Δ*t*/*h* ^2^.

From the theoretical analysis given in this paper and the numerical results shown in this section, we come to the following remark: the proposed scheme is efficient and reliable.

## 6. Concluding Remarks

In this paper, an unconditionally stable splitting scheme has been proposed to solve the nonlinear option pricing model with transaction costs. This method can be viewed as a “limit” version of LOD-Backward Euler method. This “limit” property that all subproblems are one-dimensional allows us to solve the discrete equation explicitly. As a consequence, this method is computationally efficient. The theoretical analysis carried out in this paper shows that this method is unconditionally stable, monotone, and positivity preserving. We also present several numerical experiments in which the vanilla call option and the European butterfly spread are considered. The theoretical analysis presented and the numerical results shown in this paper confirm that the proposed scheme here is efficient and reliable.

## Figures and Tables

**Table 1:** Convergence results for the scheme (45) for the transaction costs model together with a vanilla call option, where *a* = 0.015.

*M*	*N*	Value	Difference	Ratio
50	50	8.391857	—	—
100	200	8.416984	0.025127	—
200	800	8.423089	0.006105	4.115806
400	3200	8.424567	0.001487	4.105581

**Table 2:** Convergence results for the scheme (45) and the nonstandard scheme proposed in [16] for the transaction costs model together with a butterfly spread, where *a* = 0.05.

*M*	*N*	*E*_∞_^Sp^	Ratio	*E*_∞_^Ns^	Ratio
20	10	8.205076*e* − 002	—	8.209482*e* − 002	—
40	40	1.753266*e* − 002	4.679881	1.776302*e* − 002	4.621670
80	160	4.409681*e* − 003	3.975947	4.857916*e* − 003	3.656510
160	640	1.127720*e* − 003	3.910262	2.229942*e* − 003	2.178494
